# A perspective on the EANM procedure guidelines for radionuclide therapy with ^177^Lu-labelled PSMA-ligands

**DOI:** 10.1007/s00259-021-05400-5

**Published:** 2021-05-13

**Authors:** Germo Gericke

**Affiliations:** Advanced Accelerator Applications, a Novartis company, 1204 Geneva, Switzerland

Dear Sir,

As the development sponsor of [^177^Lu] Lu-PSMA-617 (^177^Lu-PSMA-617) for treatment of prostate cancer, Advanced Accelerator Applications (AAA), a Novartis company, was recently approached to comment on the ‘EANM procedure guidelines for radionuclide therapy with ^177^Lu-labelled PSMA-ligands (^177^Lu-PSMA-RLT)’ by Clemens Kratochwil and colleagues, published in the journal [1]. Queries included whether data obtained with ^177^Lu-PSMA-617 can be applied to other PSMA-targeting radioligand therapies for clinical decision-making, regulatory assessment or other purposes.

The authors of the EANM procedure guidance discuss various aspects, including radiopharmaceutical application, dosimetry, safety and efficacy.

Under ‘Radiopharmaceutical’ (page 2538), the authors provide a recommendation for exchangeable application of ^177^Lu-PSMA-617 and PSMA-I&T (imaging and therapy), based on ‘comparable biodistribution’. It should be noted that this statement is not supported by any comparative trial. In fact, AAA is not aware of any prospective trial of PSMA-I&T. Considering the inherent variability of patient populations, clinical and analytical protocols and small samples, the available, mostly retrospective, analyses and comparisons of data gathered outside clinical trials do not support a clinical comparison of biodistribution.

Under ‘Safety’ (page 2541), the authors conclude, ‘In summary, data indicate a favourable safety profile for ^177^Lu-PSMA-RLT’, which suggests a generic safety profile across all PSMA-targeting RLTs. To my knowledge, there are no clinical data available to support a comparison or generalization across various PSMA-targeting molecules.

Under ‘Efficacy’ (page 2541), the authors state ‘available data do not indicate differences in efficacy’. While this statement is always true in the absence of adequate data, it can easily be misunderstood as clinical guidance and is not appropriate for a procedure guidance. Limited retrospective data from mostly small case series of patients treated outside clinical trials do not provide sufficient scientific basis for an assessment of similarity or difference in efficacy.

Advancing the RLT platform in a credible manner depends on adherence to scientific standards, precise interpretation of available information, or lack thereof, and timely updates as new information becomes available. These scientific standards underlie both clinical guidance and regulatory assessment and available data do not support generalization of data obtained with ^177^Lu-PSMA-617 to other, structurally distinct PSMA-targeted therapeutics. The preamble statement ‘These guidelines are intended to assist practitioners in providing appropriate nuclear medicine care for patients’; the purpose statement: ‘Providing an expert consensus in favour of performing these treatments by reasonably balancing risks versus benefits’, as well as specific sections on safety and efficacy, suggest clinical guidance rather than procedural guidance. Accordingly, I respectfully request that the journal and the authors clarify the scope and aspiration of this paper and its limitations more clearly to avoid misinterpretation.

## Data Availability

Not applicable.
